# Panaxadiol Attenuates Neuronal Oxidative Stress and Apoptosis in Cerebral Ischemia/Reperfusion Injury via Regulation of the JAK3/STAT3/HIF‐1α Signaling Pathway

**DOI:** 10.1111/cns.70233

**Published:** 2025-02-17

**Authors:** Jiabin Zhou, Yu Lei, Shilin Zhang, Yuhan Liu, Dongye Yi

**Affiliations:** ^1^ Department of Neurosurgery, Union Hospital, Tongji Medical College Huazhong University of Science and Technology Wuhan Hubei Province People's Republic of China; ^2^ Wuhan University Wuhan Hubei Province People's Republic of China; ^3^ Naval Aviation University of Chinese People's Liberation Army Yantai Shandong Province People's Republic of China; ^4^ Department of Gastroenterology Hubei Provincial Hospital of Integrated Chinese and Western Medicine Wuhan Hubei Province People's Republic of China

**Keywords:** apoptosis, cerebral ischemic stroke, network pharmacology, oxidative stress, panaxadiol

## Abstract

**Background:**

Cerebral ischemic stroke (CIS) is a debilitating neurological condition lacking specific treatments. Cerebral ischemia/reperfusion injury (CIRI) is a critical pathological process in CIS.

**Purpose:**

This study aimed to explore the protective effects of panaxadiol (PD) against oxidative stress‐induced neuronal apoptosis in CIS/CIRI and its underlying mechanisms.

**Method:**

An MCAO mouse model was established to investigate the therapeutic effects of PD in vivo. Network pharmacology and molecular docking techniques were used to predict PD's anti‐CIS targets. The protective effects of PD were further validated in vitro using oxygen–glucose deprivation/reoxygenation (OGD/R)‐treated HT22 cells. Finally, core targets were verified through combined in vivo and in vitro experiments to elucidate the mechanisms of PD in treating CIS.

**Result:**

PD exhibited significant neuroprotective activity, demonstrated by restoration of behavioral performance, reduced infarct volume, and decreased neuronal apoptosis in mice. Network pharmacology analysis identified 24 overlapping target genes between PD and CIS‐related targets. The hub genes, PTGS2, SERPINE1, ICAM‐1, STAT3, MMP3, HMOX1, and NOS3, were associated with the HIF‐1α pathway, which may play a crucial role in PD's anti‐CIS effects. Molecular docking confirmed the stable binding of PD to these hub genes. Both in vitro and in vivo experiments further confirmed that PD significantly mitigates neuronal apoptosis and oxidative stress induced by CIS/CIRI.

**Conclusion:**

PD significantly counteracts CIS/CIRI by modulating the JAK3/STAT3/HIF‐1α signaling pathway, making it a promising therapeutic agent for treating CIS/CIRI.

## Graphical abstract

In this study, we demonstrated that panaxadiol alleviated oxidative stress damage in mouse brain tissue caused by CIRI through the regulation of the JAK3/STAT3/HIF‐1α signaling pathway. This study provided a theoretical basis and promising prospects for the clinical application of panaxadiol in the treatment of CIRI.

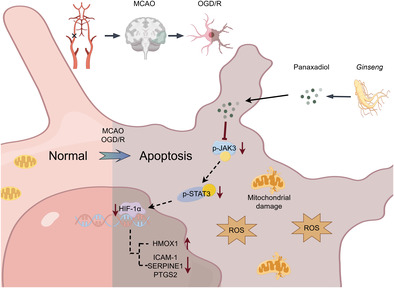

**Abbreviations**
CAT: catalase
CIRI: cerebral ischemia–reperfusion injury
CIS: cerebral ischemic stroke
ELISA: enzyme‐linked Immunosorbent Assay
MCAO: middle cerebral artery occlusion
MDA: malondialdehyde
OGD/R: oxygen–glucose deprivation/reoxygenation
PD: panaxadiol
ROS: reactive oxygen species
SOD: superoxide dismutase

## 1 Introduction

Cerebral ischemic stroke (CIS) is a common cerebrovascular disease, especially in aging populations, characterized by high disability, recurrence, and mortality rates [1]. CIS arises from arterial obstruction, causing ischemia, hypoxic damage to the local brain tissue, and subsequent neurological dysfunction [2]. Temporary or persistent insufficient blood flow leads to irreversible neuronal damage in the core area of brain tissue infarction [3], while the neurons in the surrounding ischemic penumbra experience relative hypo‐perfusion [4]. Timely restoration of blood flow, rescuing ischemic penumbral neurons, and minimizing ischemic damage are critical for effective CIS treatment. Vascular recanalization is the primary clinical therapy for CIS, but it often induces cerebral ischemia/reperfusion injury (CIRI) [5].

CIRI is a cascade reaction involving complex pathophysiological processes, including neuronal calcium overload, oxidative stress, mitochondrial damage, and apoptosis [6, 7]. The core pathogenic mechanisms include ischemia‐hypoxia, reperfusion injury, and inflammatory response [8, 9]. Ischemic stroke causes a rapid decrease in cerebral blood flow, leading to neuronal hypoxia, energy deficiency, and cell death [10]. During the reperfusion phase, neurons undergo metabolic changes generating excessive free radicals, causing oxidative stress, and aggravating neuron death [11, 12]. This is accompanied by the release of numerous inflammatory factors, causing an immune‐inflammatory response and further aggravating brain tissue damage [13, 14]. The pathophysiological process of CIS/CIRI is also related to multiple signaling pathways, among which the JAK3/STAT3/HIF‐1α signaling pathway plays an important role and is involved in inflammatory response [15], oxidative stress, and apoptosis. Ischemia–reperfusion injury overactivates the JAK3/STAT3 signaling pathway with phosphorylated JAK3 promoting STAT3 phosphorylation as well as its nuclear translocation, and cytokines (IL‐6, TNF‐α) expression, exacerbating inflammatory response. Hypoxia induces the HIF‐1α signaling pathway to upregulate pro‐inflammatory factors and increase cerebral vascular permeability, further aggravating tissue damage [16]. Currently, effective drugs for the treatment of CIS/CIRI are limited, highlighting the need for novel therapies to reduce neuronal damage and preserve neurological function in patients with CIS.

In recent years, Chinese herbal remedies and their natural medicinal compounds have gained attention for their low toxicity, multiple targets, and accurate efficacy, and have been widely used in disease prevention and treatment [17]. Ginseng, an important traditional Chinese medicine, is commonly used to treat stroke because of its strong tonic effect [18, 19]. Panaxadiol (PD), a triterpenoid saponin compound, has shown antioxidative [20], anti‐tumor [21], immune‐modulating [22], and neuroprotective properties [23, 24]. Prior studies have demonstrated its neuroprotective effects against neuronal damage induced by amyloid β‐protein deposition in Alzheimer's disease. However, the efficacy and mechanism of action of PD in CIRI remain unclear [25].

Network pharmacology is a comprehensive discipline integrating pharmacology, systems biology, and computer science [26, 27]. This provides tools for exploring the mechanisms of traditional Chinese medicine in the treatment of diseases [28, 29]. This study systematically explored the effects and potential mechanisms of action of PD in the treatment of CIS/CIRI by combining network pharmacological analyses, molecular docking, and experimental verification. We verified the therapeutic potential of PD on CIS/CIRI using the mouse MCAO model and an HT22 cells OGD/R model. We predicted possible molecular mechanisms using network pharmacology analysis and molecular docking methods and identified potential core targets of PD for CIS/CIRI treatment both in vitro and in vivo. This study provides a theoretical basis and promising prospects for PD's clinical application of CIS/CIRI treatment.

## 2 Methods and Materials

### 2.1 Network Pharmacology Analysis

#### 2.1.1 Screening of PD‐Related Targets

Three databases, PharmMapper [30], SwissTargetPrediction [31], and Super‐PRED [32] were used to identify the potential PD targets. All predicted targets were converted into Gene Symbols using the Uniprot database [33]. The species were selected as “ *Homo sapiens* ” in the Uniprot database, and duplicated and non‐human targets were removed.

#### 2.1.2 Prediction of CIS‐Associated Targets

The keyword “CIS” was used to identify disease‐associated targets from the GeneCards [34] and DisGeNET databases [35]. The gene expression profiles of mouse brain tissues (GSE30655) were downloaded from the GEO database, including seven MCAO mouse brain tissues and three sham‐surgery mouse brain tissues [36]. Gene expression profiling data were normalized by the “normalizeBetweenArrays” R package and differential expression gene analysis was performed using “limma” R package. Genes with |LogFC| > 0.58 and p.adj < 0.05 were considered differentially expressed genes (DEGs). Then, mouse DEGs were converted to human homologous genes using the “homologene” R package.

#### 2.1.3 Potential Therapeutic Targets of PD for CIS

The intersections of PD‐related targets, CIS‐associated targets, and DEGs of GSE30655 were considered potential therapeutic targets of PD for CIS.

#### 2.1.4 Protein–Protein Interaction Network Analysis and Hub Genes Acquisition

Potential therapeutic targets were imported into the STRING database to construct a protein–protein interaction network [37]. The organism was selected as “ *Homo sapiens* ,” the minimum required interaction score was set to high confidence (0.7), disconnected nodes in the network were hidden, and other parameters were set to default settings. A TSV file containing protein–protein interaction information was obtained from the STRING database and visualized using Cytoscape software [38]. Hub genes were extracted using the CytoHubba plugin of Cytoscape.

#### 2.1.5 GO and KEGG Enrichment Analyses

GO and KEGG enrichment analyses were performed using the “clusterProfiler” R package to explore the biological functions of the potential therapeutic targets of PD for CIS. Statistical significance was set at *p* < 0.05 FDR < 0.05. The chord plots of these results were generated with the “GOplot” R package. The marked HIF‐1 signaling pathway was generated by the “Pathview” R package.

#### 2.1.6 Molecular Docking

The molecular docking steps have been described in our previous study [39]. In brief, the SDF format file of PD was acquired from the PubChem database and converted into a MOL2 format file using Chem3D software. Protein structure files were downloaded from the PDB database [40]. AutoDock tools were used to pretreat the protein structure and obtain a pdbqt format file [41]. Molecular docking was performed using AutoDock Vina, and the results were visualized using PYMOL software and BIOVIA Discovery Studio software. The Heatmap of binding energy was plotted using “ggplot2” R package.

### 2.2 Behavioral Tests

#### 2.2.1 Longa Score

After 10 days of consecutive gastric gavage in mice, the Longa score was used for evaluation. The Longa scoring system 5‐point scale is defined as follows: Grade 0, no observable neurological deficits in mice; Grade 1, the inability of mice to fully extend one forelimb on the paralyzed side; Grade 2, mice circling toward the paralyzed side while crawling; Grade 3, mice leaning toward the paralyzed side while crawling; and Grade 4, mice unable to ambulate spontaneously and lose consciousness.

#### 2.2.2 Rotarod Test

Mice were first subjected to adaptive training using a rotarod apparatus. During the formal experiment, the speed of the rotating rod was increased gradually from 5 to 40 rpm, until the mice lost balance and fell off.

#### 2.2.3 Balance Beam Test

Before the formal experiment, the mice underwent a 3‐day balance beam training program. The training involved traversing a wooden beam (50 cm long, 0.6 cm wide, and 80 cm above the ground), three times a day. During testing, the number of falls and slips while crossing the beam was recorded and their mean was calculated. Behavioral scoring was conducted based on the following criteria: 0 points, mouse falls directly from the beam; 1 point, mouse lies on the beam but cannot move; 2 points, mouse falls while crawling on the beam; 3 points, mouse slips on > 50% of crawling steps while crossing the beam; 4 points, mouse slips on < 50% of crawling steps while crossing the beam; 5 points, mouse slips only once; 6 points, mouse successfully crosses the beam without slipping.

### 2.3 Reagents and Antibody

PD (B21040, CAS No. 19666‐76‐3) was purchased from Yuanye Bio‐Technology Co. Ltd. (Shanghai, China) and dissolved in DMSO. Silicon‐coated monofilament nylon suture (45‐0420) were purchased from Getimes Technology Co. Ltd. (Beijing, China). 2, 3, 5‐triphenyltetrazolium chloride Solution (G3005), Nissl Stain Solution (Toluidine Blue Method, G1436), and hematoxylin–eosin (HE) Stain Kit (G1120) were purchased from Solarbio Science & Technology Co. Ltd. (Beijing, China). Fetal bovine serum (FBS500‐H) was bought from HYCEZMBIO (Wuhan, China). Dulbecco's Modified Eagle's medium (DMEM)‐High Glucose (G4511), dimethyl sulfoxide (DMSO; GC203005), and penicillin/streptomycin (G4003) were purchased from Service Bio (Wuhan, China). IL‐1β, IL‐6, and TNF‐α ELISA Kits were purchased from Thermo Fisher Scientific (Wuhan, China). A Cell Counting Kit (CCK‐8, HYCCK8) was purchased from HYCEZMBIO (Wuhan, China). The ROS Assay Kit (G1706), Annexin V‐FITC/PI Cell Apoptosis Detection Kit (G1511), TMR (red) Tunel Cell Apoptosis Detection Kit (G1502), and JC‐1 Mitochondrial Membrane Potential Assay Kit (G1515) were purchased from Servicebio (Wuhan, China). SOD, CAT, and MDA assay kits were purchased from the Jiancheng Bioengineering Research Institute (Nanjing, China). Primary antibodies included Bcl‐2 (26593‐1‐AP, Proteintech, Wuhan, China), Bax (GB114122, Servicebio, Wuhan, China), Cleaved Caspase‐3 (25128‐1‐AP, Proteintech, Wuhan, China), NeuN (26975‐1‐AP, Proteintech, Wuhan, China), Jak3 (80331‐1‐RR, Proteintech, Wuhan, China), Phospho‐Jak3 (29101‐1‐AP, Proteintech, Wuhan, China), Phospho‐Stat3 (9131S, Cell Signaling Technology, Massachusetts, USA), Stat3 (9139S, Cell Signaling Technology, Massachusetts, USA), HIF‐1 α (66730‐1‐Ig, Proteintech, Wuhan, China), ICAM‐1 (60299‐1‐Ig, Proteintech, Wuhan, China), HMOX1 (10701‐1‐AP, Proteintech, Wuhan, China), PAI‐1 (66261‐1‐Ig, Proteintech, Wuhan, China), and PTGS2 (66351‐1‐Ig, Proteintech, Wuhan, China). Secondary antibodies included HRP‐conjugated goat anti‐rabbit IgG and anti‐mouse IgG (Servicebio, Wuhan, China).

### 2.4 Cell Culture and OGD/R Treatment

The mouse hippocampal neuronal cell line, HT22, was obtained from the Cell Culture Center, Institute of Basic Medical Sciences, Chinese Academy of Medical Sciences (Beijing, China). HT22 cells were cultured in a complete medium (DMEM‐high glucose with 10% FBS and 1% penicillin/streptomycin) at 37°C in a humidified incubator with 5% CO_2_.

HT22 cells at 80%–85% confluence underwent OGD/R treatment to replicate ischemia/reperfusion conditions. The culture medium was replaced with OGD medium (DMEM sugar‐free), and HT22 cells were exposed to hypoxia (94% N_2_, 5% CO_2_, and 1% O_2_) in a three‐gas hypoxia incubator for 4 h. HT22 cells were then cultured in a complete medium for 24 h in a standard cell culture incubator.

### 2.5 Cell Viability Assay

HT22 cells were seeded at a density of 5000 cells/well in 96‐well plates and incubated overnight for adherence. After OGD/R treatment, HT22 cells were exposed to varying concentrations of PD (0.5, 1, 2, 4, 8, 16, and 32 μM) for 24 h. Subsequently, 10 μL of CCK8 solution was added into each well, and the plates were incubated for 2 h. The absorbance at 450 nm was measured using a multifunctional microplate reader (SpectraMax iD3, USA), and cell viability was determined from the absorbance values.

### 2.6 Reactive Oxygen Species (ROS) Assay

A DCFH‐DA fluorescent probe was used to measure the intracellular ROS levels. Following OGD/R treatment and PD intervention, cells were co‐incubated with the DCFH‐DA probe for 20 min. Subsequently, the cells were washed thrice with serum‐free culture medium and observed under a fluorescence microscope for imaging.

### 2.7 Mitochondrial Membrane Potential Assay

JC‐1 staining buffer and working solutions were prepared according to the manufacturer's instructions. After washing the cells with JC‐1 staining buffer, 1 mL of DMEM and 1 mL of JC‐1 staining working solution were added to each well of a 6‐well plate. The cells were then incubated for 20 min. Following incubation, the cells were washed twice with the JC‐1 staining buffer and imaged using a fluorescence microscope.

### 2.8 Cell Apoptosis Assay

An Annexin V‐FITC/PI apoptosis detection kit combined with flow cytometry was used to detect cell apoptosis. After 24 h of OGD/R treatment and PD intervention, HT22 cells and supernatants were collected and centrifuged for 5 min. The cell pellet was resuspended in binding buffer at the appropriate dilution. FITC and PI reagents were sequentially added, and the cells were incubated in the dark for 15 min. Apoptosis detection was performed using a flow cytometer, with data collected and analyzed using CytExpert software.

### 2.9 Animal Experimental Design

Adult male BALB/c mice (8 weeks old, 22–23 g) were obtained from SiPeiFu (Beijing, China) and housed in a specific pathogen‐free (SPF) and temperature‐controlled environment. The experimental protocol was approved by the Laboratory Animal Ethics Committee (approval number: ZN2023094). All experiments were performed in accordance with the animal experimental guidelines of Zhongnan Hospital of Wuhan University.

Based on previous research, a mouse model of CIRI was developed by intraluminal occlusion of the right middle cerebral artery (MCA) [42]. In summary, the mice were anesthetized using sodium pentobarbital (50 mg/kg). To induce MCA occlusion, a silicon‐coated monofilament nylon suture was inserted into the internal carotid artery and advanced to block the MCA. After 40 min, the filament was retracted to allow for reperfusion. During surgery, the mice's body temperature was maintained at 37.5°C using a thermostatic heating blanket. Relative cerebral blood flow (rCBF) was monitored using laser speckle contrast imaging (SIM BFI HR Pro; Simopto, Wuhan, China) during occlusion and reperfusion.

**FIGURE 1:**
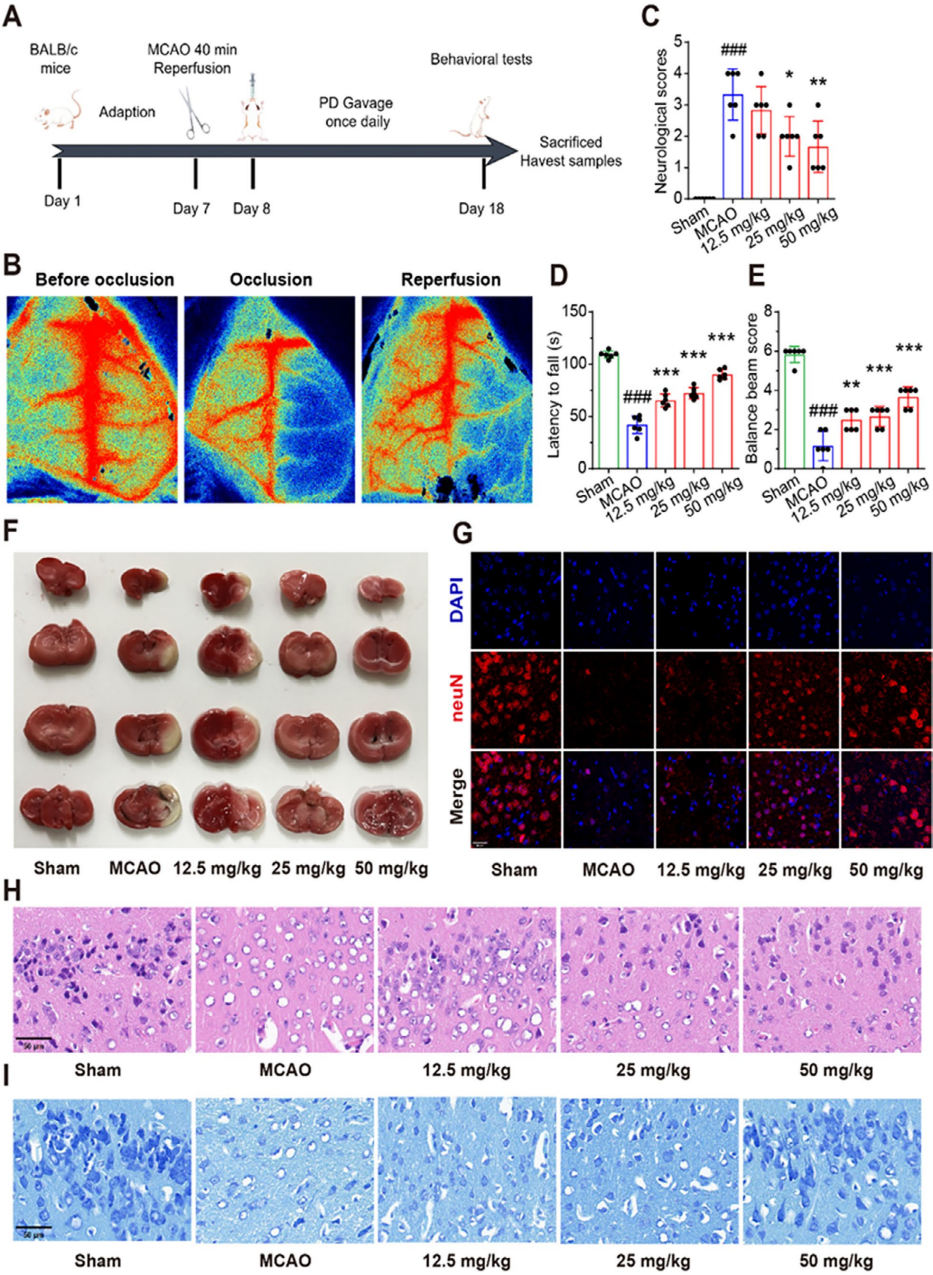
PD exhibits significant neuroprotective effects in MCAO models of cerebral ischemic stroke. (A) The animal experimental design in this experimental. (B) Representative laser speckle images. (C) Longa score. (D) The rotarod test. (E) Balance beam test. (F) TTC staining. (G) Representative fluorescence pictures of NeuN. (H) Representative H&E staining images. (I) Representative Nissl staining images. # # #*p* < 0.001 as compared with Sham group; *p < 0.05, **p < 0.01, ***p < 0.001 as compared with MCAO group.

Based on the prior studies, we selected PD with three different concentrations for animal experiments [43, 44]. The mice were randomly allocated to five groups, each consisting of six mice: sham group, MCAO group, and three PD‐treated groups receiving low (10 mg/kg), medium (20 mg/kg), or high (30 mg/kg) concentrations. Following MCAO, the mice received their respective PD doses or saline (for the sham and MCAO groups) for 10 days. Behavioral assessments were conducted after the treatment period. Subsequently, all mice were humanely euthanized for further experiments (Figure 1A).

### 2.10 2, 3, 5‐Triphenyltetrazolium Chloride (TTC) Staining Assay

After slicing the mouse brain tissue, the slices were immersed in TTC staining solution and incubated at 37°C for 15 min, flipping every 5 min to ensure uniform staining. Following staining, the samples were washed with PBS, observed, and photographed.

### 2.11 Immunofluorescent Staining Assay

The brain tissue slices were washed three times with PBST for 5 min each and then incubated in a blocking solution for 2 h. After blocking, the slices were incubated with anti‐NeuN antibody overnight at 4°C. The following day, the slices were washed three times with PBST for 5 min, followed by a 2‐h incubation with goat anti‐rabbit IgG. The slices were then stained with DAPI for 10 min, washed with PBST, and photographed.

### 2.12 HE Staining Assay

The harvested brain tissue samples were fixed, embedded in paraffin, and sectioned. Deparaffinization was performed twice in xylene for 5 min each. The sections were then rehydrated using a series of ethanol gradients (100%, 95%, 85%, 75%), with each step lasting 3 min, followed by soaking in distilled water for 2 min. Hematoxylin staining was performed for 15 min, and excess stain was removed by rinsing with distilled water. Differentiation was carried out for 30 s, followed by two 3‐min rinses with tap water. The sections were then stained with eosin solution for 1 min, excess stain was removed, and rapid dehydration was performed. This included brief immersion in 75%, 85%, 95%, and 100% ethanol for 2–3 s each, followed by immersion in fresh 100% ethanol for 1 min. Finally, the sections were cleared twice in xylene for 1 min each time, mounted with neutral resin, and observed under a microscope.

### 2.13 Nissl Staining Assay

Paraffin‐embedded brain tissue sections were deparaffinized in water. The slides were stained in a solution of toluidine blue at 60°C for 30 min, briefly rinsed in distilled water, and rapidly differentiated in 95% ethanol. The slides were then dehydrated in absolute ethanol, cleared in xylene, and mounted with neutral resin. The slides were then observed under a microscope.

### 2.14 TUNEL Staining Assay

A TUNEL staining kit was used to identify apoptotic neurons following the manufacturer's guidelines. Initially, tissue sections were deparaffinized, hydrated, and treated with proteinase K for 20–30 min to enhance permeability. Subsequently, the sections were incubated in a membrane permeabilization solution for 20 min, followed by a 10‐min co‐incubation with equilibration buffer at room temperature. The TUNEL reaction mixture was evenly applied to the sections and incubated for 1 h at 37°C under light‐protected conditions. Finally, the sections were stained with DAPI, mounted, and imaged using a fluorescence microscope.

### 2.15 Western Blotting

The protein lysis buffer used in this study consisted of radiommunoprecipitation assay (RIPA; HYC00825, HYCEZMBIO, Wuhan, China), phenylmethylsulfonyl fluoride (PMSF; HYP112, HYCEZMBIO, Wuhan, China), and a protease inhibitor cocktail (HYP111‐5, HYCEZMBIO, Wuhan, China). Total protein was extracted from HT22 cells and brain tissue using this buffer, followed by centrifugation to collect the supernatant. Protein concentrations were determined using a BCA kit (P0009; Beyotime, Shanghai, China). Subsequently, 30 μg of protein samples were electrophoresed on a 4%–20% MOPS gel (M00930, Genscript, Nanjing, China) and transferred to PVDF membranes (IPVH00010, Merck Millipore, Ireland). Membranes were blocked using the Fastest Blocking Buffer (HYC00811, HYCEZMBIO, Wuhan, China) for 5 min at room temperature, and incubated overnight at 4°C with the primary antibody. Following three washes with TBST (G0004; Servicebio, Wuhan, China), the membranes were incubated with a secondary antibody for 1 h at room temperature. Protein expression on the membranes was visualized using Odyssey XF imaging equipment (LI‐COR, USA) after a 1‐min immersion in West ECL solution (HYC0316, HYCEZMBIO, Wuhan, China). The relative protein band densities were quantified using ImageJ software.

### 2.16 Enzyme‐Linked Immunosorbent Assay (ELISA)

An ELISA kit was used to measure IL‐1β, IL‐6, TNF‐α, SOD, CAT, and MDA levels in HT22 cell supernatants and mice ischemic cortex according to the manufacturer's instructions. In brief, samples were added to antigen‐pre‐coated plates, sealed, and incubated at 37°C for 2 h. After washing, antibodies were added to the plates and incubated for 1 h. Subsequently, enzyme‐labeled reagents and chromogenic substrates were sequentially added, followed by a 30‐min incubation in the dark. Finally, the reaction was terminated, and a multifunctional microplate reader (SpectraMax iD3, USA) was used to detect absorbance at 450 nm.

### 2.17 Statistical Analysis

All data are presented as mean ± standard deviation (SD) and analyzed using GraphPad Prism 8 software. All data conform to the normal distribution using the Shapiro–Wilk normality test. Unpaired t‐tests or one‐way analysis of variance (ANOVA) were employed to evaluate statistical significance, with *p* < 0.05 considered significant.

## 3 Results

### 3.1 PD Exerted Significant Protective Roles in Improving Neurological Dysfunction and Cerebral Neuron Loss in MCAO Mice

A mouse MCAO model was successfully constructed to simulate the cerebral ischemia/reperfusion process. Laser speckle imaging was used to detect changes in blood flow in the MCA. As shown in Figure 1B, under normal conditions, blood flow in the blood vessels on both sides of the mouse brain appeared smooth. After MCA occlusion, blood flow on the right side of the mouse brain almost disappeared. Upon reperfusion, the blood supply to the right side of the brain was partially restored. To investigate whether PD pretreatment provides neuroprotective benefits against neural damage induced by MCAO, we conducted behavioral tests and TTC staining in MCAO mice 10 days after PD intervention and analyzed the effect of PD on improving neurological function and cerebral infarct volume. The results demonstrated that PD pretreatment significantly improved neurological deficits (Figure 1C) and motor function (Figure 1D,E) in MCAO mice and decreased the cerebral infarct area (Figure 1F). Additionally, neuronal loss in MCAO mice was determined across different PD administration groups. In the sham group, NeuN‐positive dots were vividly stained and well organized. In contrast, the MCAO group exhibited an almost complete absence of NeuN markers within the infarct regions. Notably, PD treatment effectively restored the proportion of NeuN‐positive spots, particularly in the medium‐ and high‐concentration groups, indicating potential therapeutic effects (Figure 1G). The HE staining results showed that the brain tissue of the sham group exhibited a clear structure, normal cell morphology, uniform cell staining, and centrally located cell nuclei. In the MCAO group, the infarcted cerebral tissue showed a disrupted structure with a disorganized cell arrangement, reduced neuronal count, abnormally enlarged cells, nuclear shrinkage, and increased intercellular spacing. The morphology of the neurons in the PD group was significantly improved compared to that of the MCAO group, showing intact cell structure, minimal nuclear shrinkage, and an increased number of normal cells (Figure 1H). Nissl staining was performed to observe neuronal morphology. In the sham group, neurons exhibited normal morphology with orderly cell arrangement and abundant Nissl bodies. Conversely, the MCAO group, exhibited abnormal neuronal morphology, including a reduced number of cells, disorganized arrangement, unclear outlines, and significantly fewer Nissl bodies. In comparison, the cerebral tissue in the PD group showed significant improvement, characterized by uniform cell staining and an increased number of Nissl bodies (Figure 1I). These findings suggest that PD plays a significant protective role against MCAO‐induced neuronal loss. Furthermore, pretreatment with PD at a dose of 50 mg/kg proved to be the most effective in improving MCAO‐induced neurological deficits.

### 3.2 Network Pharmacology Analysis Results

#### 3.2.1 Acquisition of PD Targets for CIS Treatment

**FIGURE 2:**
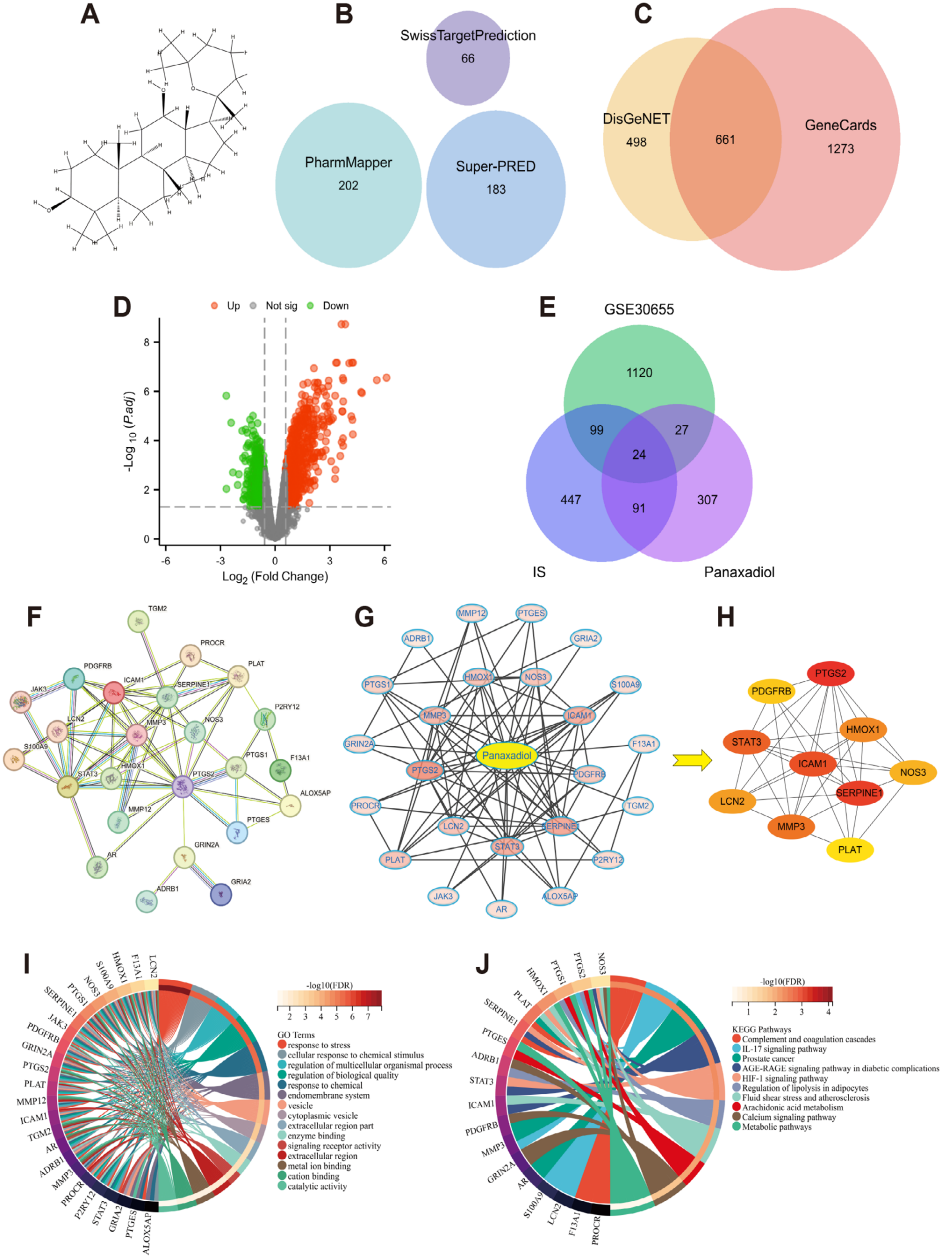
Pharmacological network analysis of PD Treatment for CIS. (A) The molecular structure of the PD. (B) From 3 databases, a total of 451 targets related to PD were obtained. (C) Obtained 661 disease genes from 2 disease databases. (D) The volcano plot. (E) A total of 24 potential therapeutic targets for treating CIS were identified. (F) The PPI network graph. (G) The PD‐targets network diagram. (H) Using the Cytohubba plugin in Cytoscape software, the top 10 hub genes identified using the MCC method. (I) The diagram of GO enrichment analysis of PD‐CIS genes. (J) The diagram of the KEGG enrichment pathway of PD‐CIS genes.

The molecular structure of PD was retrieved from PubChem database (Figure 2A). A total of 451 PD‐related targets were obtained from the three databases: 66 from the SwissTargetPrediction database, 202 from the PharmMapper database, and 183 from the Super‐PRED database (Figure 2B). Additionally, 1159 CIS‐associated targets were extracted from the DisGeNET database, and 1934 targets from the GeneCards database. The Venn plot shows 661 common targets between the two disease‐associated databases (Figure 2C). As shown in Figure 2D, we screened 1444 DEGs from the GSE30655 dataset, and 1270 human homologous DEGs were obtained after human‐mouse homologous gene conversion. As a result, 24 intersected targets were identified as the potential therapeutic targets of PD for CIS (Figure 2E).

#### 3.2.2 Protein–Protein Interaction (PPI) Network and Screening of Hub Genes

To further identify the core targets for PD treatment of CIS, a PPI network of 24 potential therapeutic targets was obtained from the STRING database (Figure 2F). A PD‐target network diagram was then generated using the Cytoscape software (Figure 2G), comprising 25 nodes (24 targets and 1 drug) and 88 edges. The targets were arranged according to their degree values, with darker node colors indicating higher degree values. Furthermore, we extracted the top 10 hub genes based on the Maximum Clique Centrality (MCC) values using the cytohubba plugin in Cytoscape (Figure 2H). The hub genes were: PTGS2, SERPINE1, ICAM‐1, STAT3, MMP3, HMOX1, NOS3, LCN2, PDGFRB, and PLAT.

#### 3.2.3 GO and KEGG Enrichment Analyses

**FIGURE 3:**
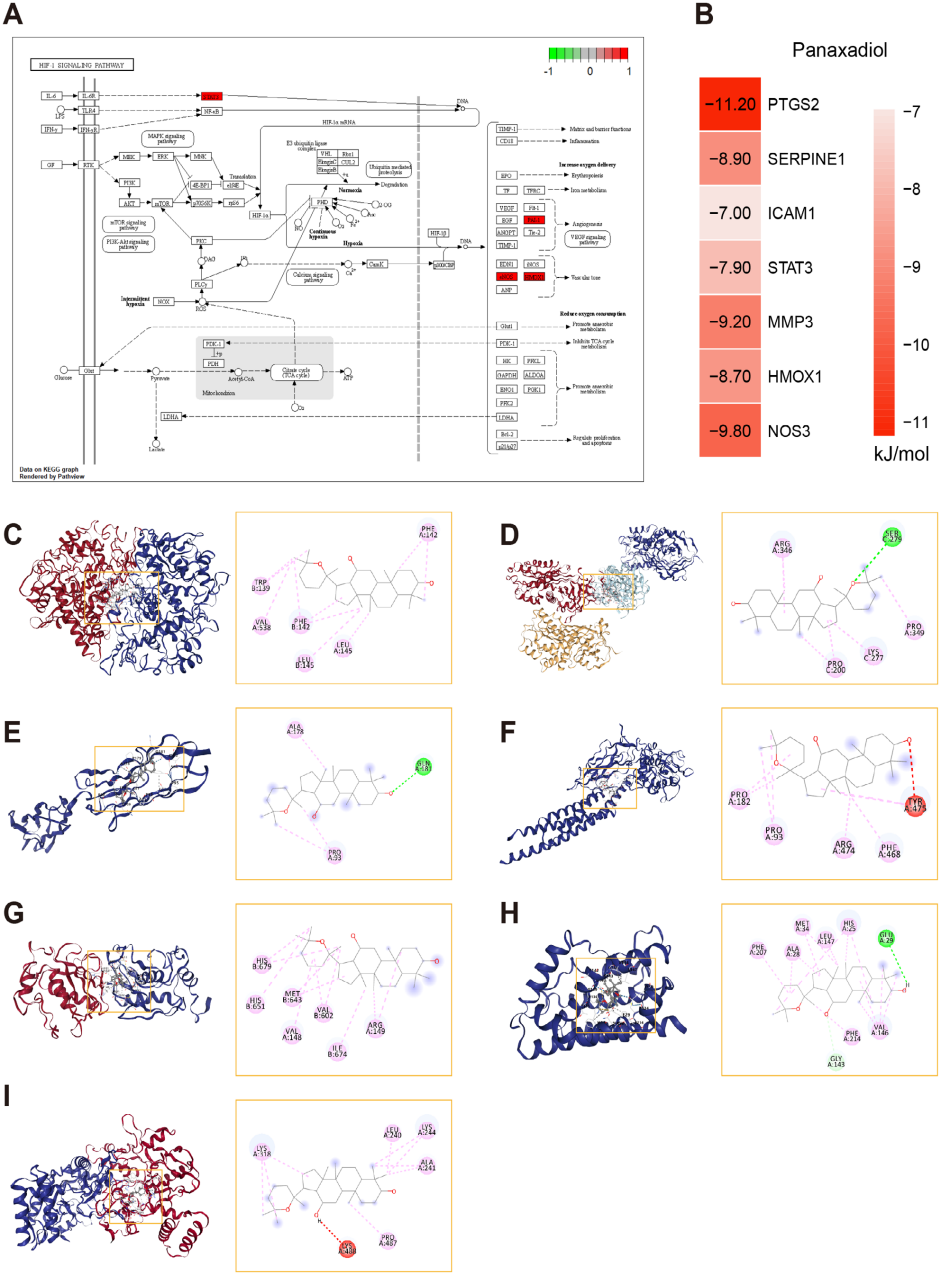
Molecular docking of PD and targets. (A) The HIF‐1 signaling pathway map from the KEGG database (B) Heatmap of PD and 7 hub genes. (C) PTGS2. (D) SERPINE1. (E) ICAM‐1. (F) STAT3. (G) MMP3. (H) HMOX1. (I) NOS3.

To demonstrate the biological functions and mechanisms of potential therapeutic targets of PD in CIS, we performed GO and KEGG enrichment analyses. A total of 735 GO entries were enriched, including 878 biological process (BP) entries, 55 cellular component (CC) entries, and 76 molecular function (MF) entries. The targets in the BP category were mainly enriched in response to stress, cellular response to chemical stimulus, regulation of multicellular organismal process, regulation of biological quality, and response to chemicals (Figure 2I). For CC, the targets were mainly involved in the endomembrane system, vesicles, cytoplasmic vesicles, extracellular regions, and enzyme binding. For MF, the targets were associated with signaling receptor activity, extracellular region, metal ion binding, cation binding, and catalytic activity. Additionally, 13 pathways were enriched in the KEGG enrichment analyses. The targets were mainly enriched in the complement and coagulation cascades, IL‐17 signaling pathway, prostate cancer, AGE‐RAGE signaling pathway in diabetic complications, and the HIF‐1 signaling pathway (Figure 2J). Furthermore, we downloaded the HIF‐1 signaling pathway map from the KEGG database and found that STAT3, SERPINE1 (PAI‐1), eNOS (NOS3), and HMOX1 were enriched in this pathway (Figure 3A).

#### 3.2.4 Molecular Docking

The top seven hub targets obtained from the cytoHubba plugin (PTGS2, SERPINE1, ICAM‐1, STAT3, MMP3, HMOX1, and NOS3) were selected for molecular docking with PD. The binding energy indicated the binding activity between the ligand and target, with lower binding energies, indicating stronger binding activities. PD exhibited a good binding activity to all selected targets (the results of binding energy were lower than −5.0 kJ/mol) (Figure 3B). The 3D and 2D diagrams of the binding modes between PD and the seven targets are shown in Figure 3C–I.

### 3.3 PD Alleviated OGDR‐Induced Oxidative Stress Injury in HT22 Cells

**FIGURE 4:**
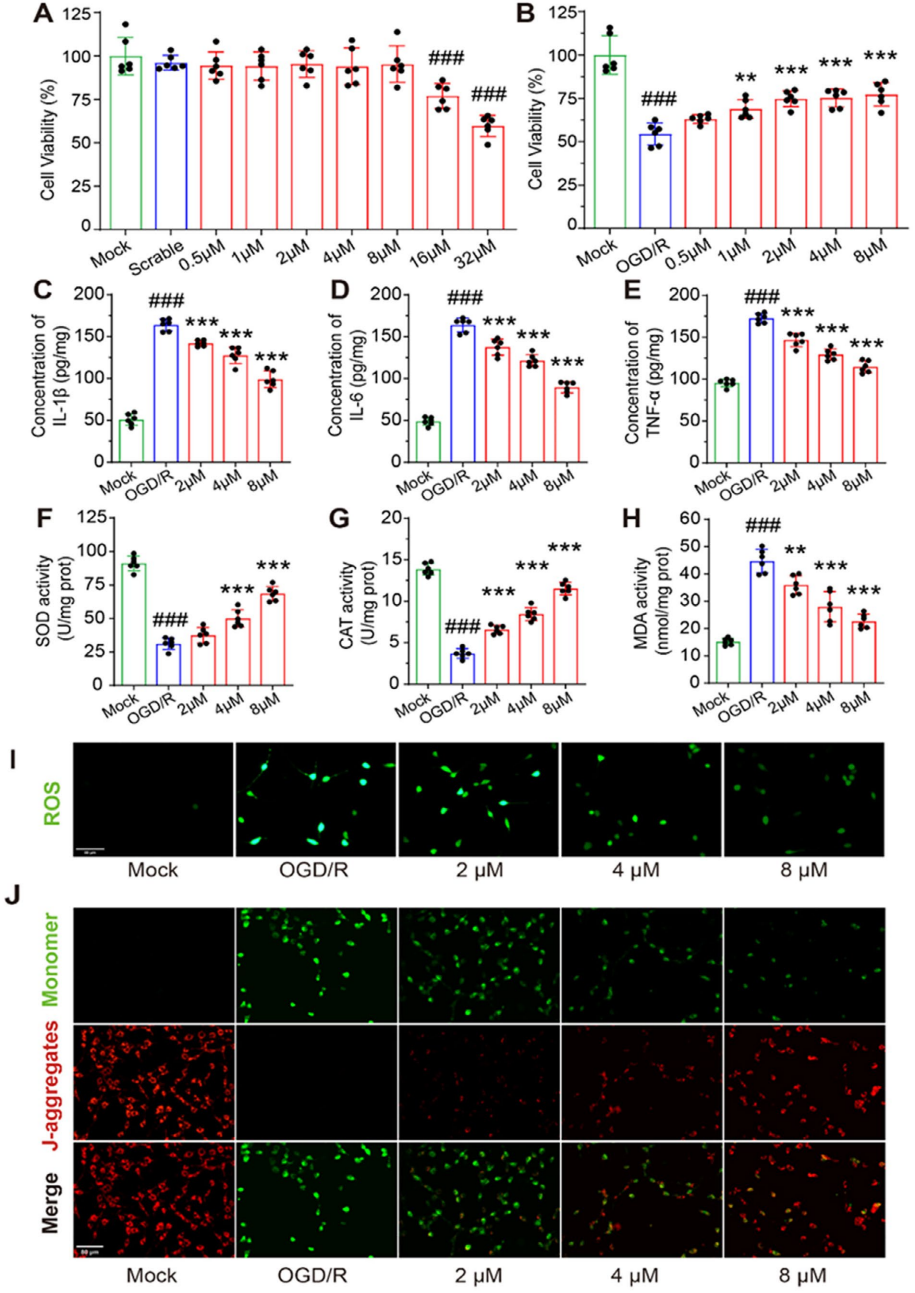
PD alleviates OGD/R induced apoptosis and oxidative stress in HT22 neurons. (A and B) Bar graph of the relative cell viability in each group. (C–E) Bar graphs of the levels of IL‐1β, IL‐6, and TNF‐α in each group. (F–H) Bar graphs of the levels of SOD, CAT, and MDA in each group. (I) Representative images for cellular ROS. (J) Representative images of JC‐1 staining. # # #*p* < 0.001 as compared with Mock group; **p < 0.01, ***p < 0.001 as compared with OGD/R group.

Mouse hippocampal HT22 cells were used to construct an OGD/R cell model to explore the neuroprotective effects of PD in vitro. First, we treated HT22 cells with different concentrations of PD (0.5–32 μM) for 24 h to evaluate their cytotoxicity. Our results showed that PD had minimal cytotoxicity on HT22 cells at concentrations of 8 μM and below (Figure 4A). Further experiments demonstrated that cell viability of HT22 cells in the OGD/R group was significantly decreased compared to that in the MOCK group. However, PD treatment exhibited significant protective effects on HT22 cells at concentrations of 2, 4, and 8 μM (Figure 4B). Pro‐inflammatory cytokines are known to play a crucial role in CIRI, contributing to the activation of the inflammatory response and cell death. In this experiment, the levels of pro‐inflammatory cytokine (IL‐1β, IL‐6, and TNF‐α) were significantly increased in the cell supernatants of OGD/R groups. Notably, PD treatment effectively reversed the increase in these inflammatory cytokines (Figure 4C–E). We also found that PD significantly increased the levels of antioxidant enzymes (SOD and CAT) in OGD/R‐treated HT22 cells (Figure 4F,G) and decreased the level of the lipid peroxide product MDA (Figure 4H). Changes in the levels of antioxidant enzymes and MDA often influence intracellular ROS levels. Therefore, we further measured intracellular ROS levels in HT22 cells using a DCFH‐DA fluorescent probe. Compared to the MOCK groups, the intracellular ROS levels in the OGD/R groups were significantly increased, whereas the PD treatment significantly reduced the increase in intracellular ROS levels in HT22 cells induced by OGD/R (Figure 4I). Mitochondrial function is closely related to ROS levels. Thus, we measured the mitochondrial membrane potential of HT22 cells using a JC‐1 fluorescent probe. At a high mitochondrial membrane potential, JC‐1 forms aggregates (J‐aggregates), and emits red fluorescence. Conversely, JC‐1 exists as monomers and emits green fluorescence at a low mitochondrial membrane potential. Compared with the MOCK groups, HT22 cells showed a significant decrease in mitochondrial membrane potential in the OGD/R groups, which was remarkably reversed by PD treatment (Figure 4J). In summary, PD promoted the levels of SOD and CAT, inhibited OGD/R‐induced increase of ROS levels in HT22 cells, and alleviated mitochondrial damage. The neuroprotective effect of PD on HT22 cells was found to be concentration‐dependent.

### 3.4 PD Inhibited OGD/R‐Induced HT22 Cells Apoptosis by Modulating the JAK3/STAT3/HIF‐1α Signaling Pathway

**FIGURE 5:**
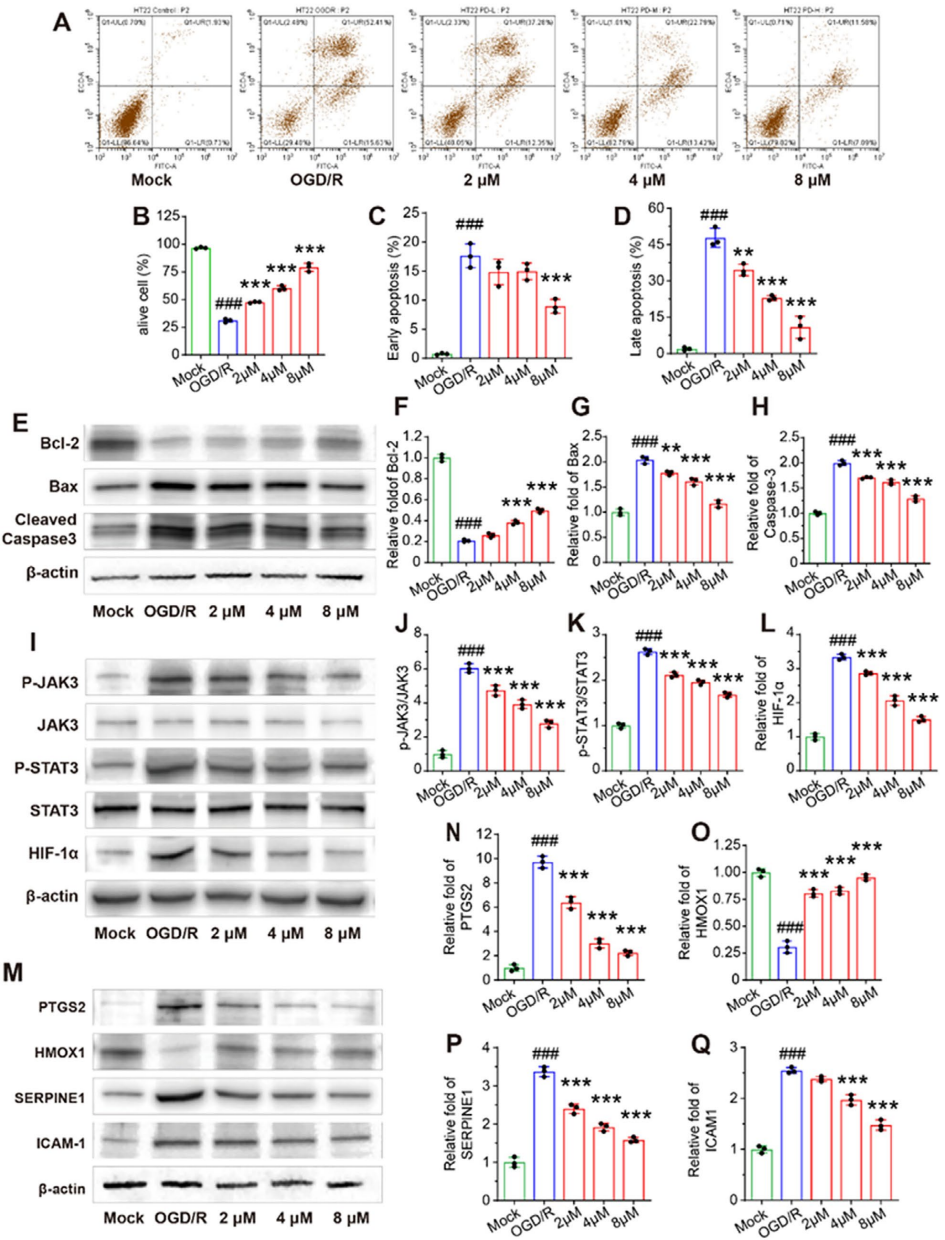
PD inhibits cellular oxidative stress and apoptosis in vitro by modulating the JAK3/STAT3/HIF‐1α signaling pathway. (A) The effects of PD on the apoptotic rate based on flow cytometry. (B–D) Flow cytometry statistics: Ratio of alive, early apoptotic, and late apoptotic HT22 cells. (E) Western blot assay of Bcl‐2, Bax, and Cleaved‐Caspase‐3. (F–H) Quantitative analysis of the bands reveals Bcl‐2, Bax, and Cleaved‐Caspase‐3 levels. (I) Representative bands of P‐JAK3/JAK3, P‐STAT3/STAT3, and HIF‐1α levels. (J–L) Quantitative analysis of the bands reveals P‐JAK3/JAK3, P‐STAT3/STAT3, and HIF‐1α levels. (M) The protein levels of PTGS2, HMOX1, SERPINE1, and ICAM‐1. (N–Q) Quantitative analysis of the bands reveals PTGS2, HMOX1, SERPINE1, and ICAM‐1 levels. # # #*p* < 0.001 as compared with Mock group; **p < 0.01, ***p < 0.001 as compared with OGD/R group.

Flow cytometry was used to assess the level of apoptosis in HT22 cells. The results revealed that, compared to the MOCK groups, HT22 cells in the OGD/R groups exhibited an increase in the rate of cell apoptosis, while the PD groups demonstrated a strong protective effect on OGD/R‐induced HT22 cells (Figure 5A–D). Specifically, PD administration significantly reduced both early and late apoptosis in HT22 cells in a concentration‐dependent manner, with a more pronounced decrease in late apoptosis (Figure 5C,D). Several apoptosis‐associated markers in HT22 cells were also evaluated. Compared to the MOCK groups, the OGD/R groups showed a decrease in Bcl‐2 expression, and an increase in the expression of Bax and cleaved Caspase‐3. In contrast, compared to the OGD/R groups, the PD groups showed increased levels of Bcl‐2 and decreased levels of Bax and cleaved Caspase‐3 (Figure 5E–H).

Based on the results of hub targets and key signaling pathways predicted by network pharmacology analysis, we examined the expression of certain proteins. Compared to the MOCK groups, the protein levels of PTGS2, SERPINE1, and ICAM‐1 were significantly increased, while the expression of HMOX1 was decreased in the OGD/R groups (Figure 5M–Q). PD treatment reversed these changes in protein expression levels induced by OGD/R in a dose‐dependent manner. Additionally, we evaluated the changes in protein levels of the JAK3/STAT3/HIF‐1α signaling pathway (Figure 5I–L). OGD/R groups showed elevated protein levels of phosphorylate‐JAK3, phosphorylate‐STAT3, and HIF‐1α. However, PD treatment significantly suppressed the OGD/R‐induced increase in these proteins. These findings indicate that PD reduced OGD/R‐induced apoptosis in HT22 cells, and its neuroprotective effects may be related to the regulation of hub genes (PTGS2, HMOX1, SERPINE1, and ICAM‐1) as well as the JAK3/STAT3/HIF‐1α signaling pathway.

### 3.5 PD Inhibited Neuronal Oxidative Stress and Apoptosis Through Regulating JAK3/STAT3/HIF‐1α Signaling Pathway in MCAO Mice

**FIGURE 6:**
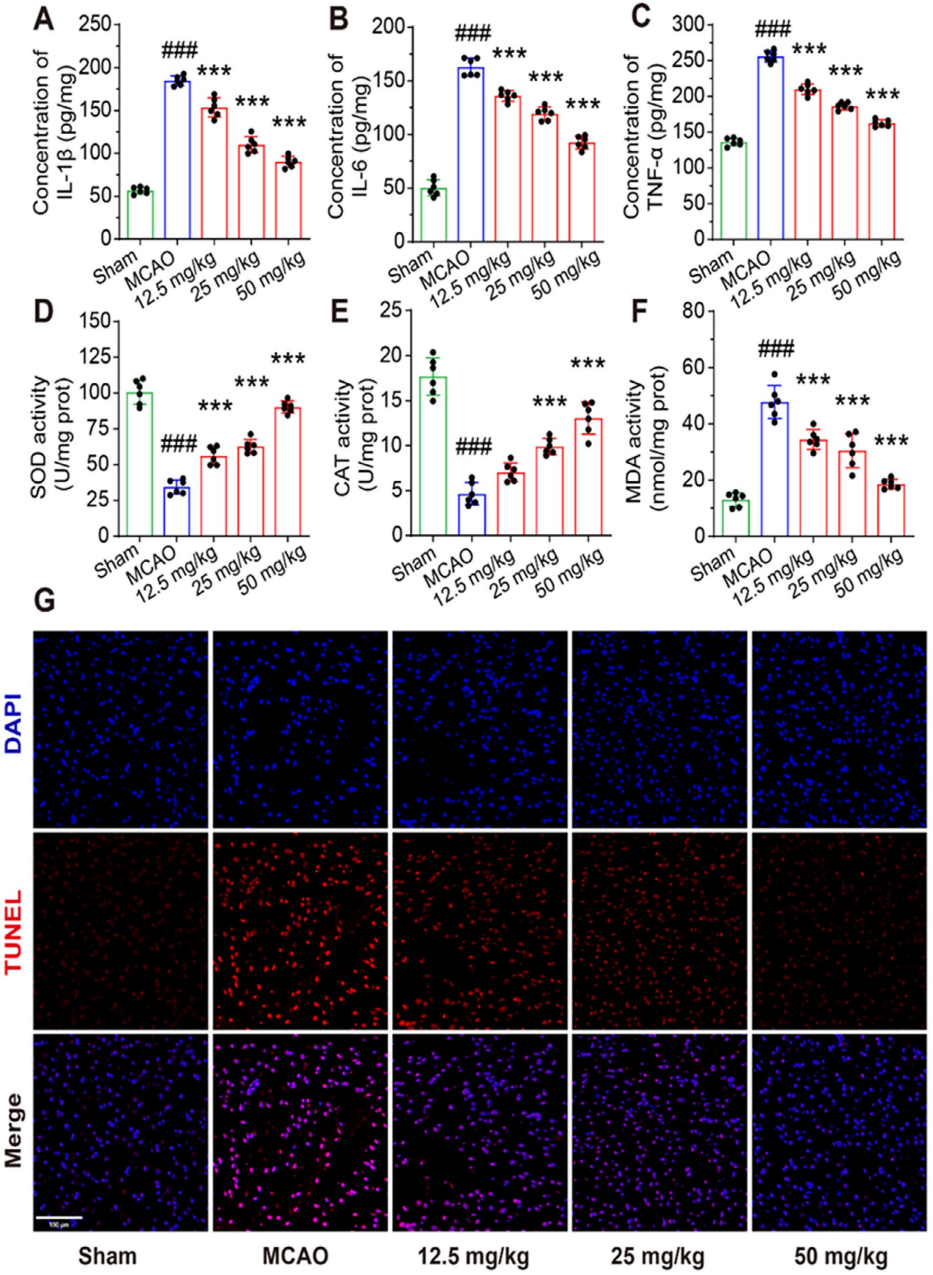
PD inhibits oxidative stress and neuronal apoptosis in MCAO mice.(A‐C) Bar graphs of the levels of IL‐1β, IL‐6, and TNF‐α in each group. (D–F) Bar graphs of the levels of SOD, CAT, and MDA in each group. (G) Representative images of TUNEL staining. # # #*p* < 0.001 as compared with Sham group; ***p < 0.001 as compared with MCAO group.

**FIGURE 7:**
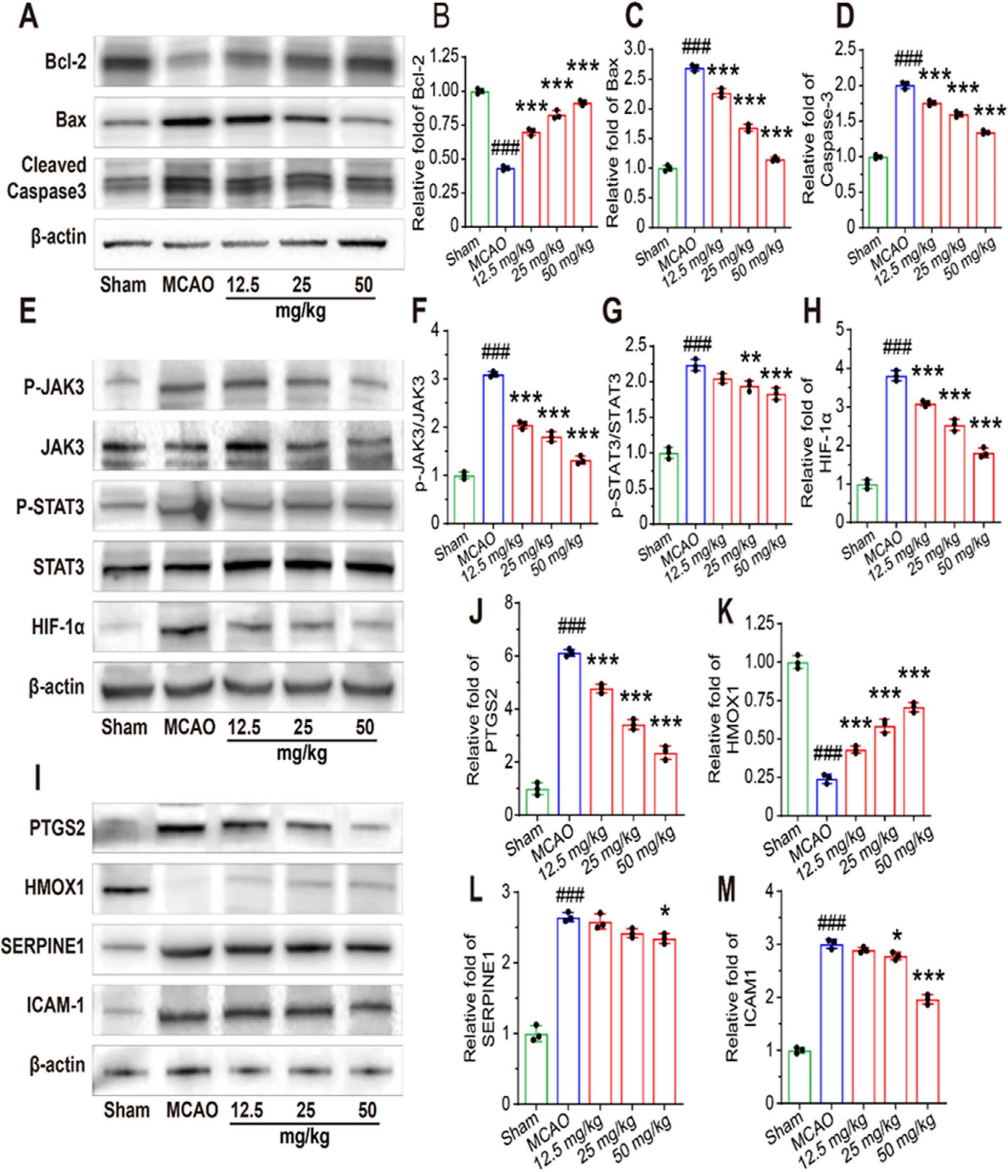
PD inhibits neuronal apoptosis and oxidative stress in mice by modulating the JAK3/STAT3/HIF‐1α signaling pathway. (A) Western blot assay of Bcl‐2, Bax, and Cleaved‐Caspase‐3. (B–D) Quantitative analysis of the bands reveals Bcl‐2, Bax, and Cleaved‐Caspase‐3 levels. (E) Representative bands of P‐JAK3/JAK3, P‐STAT3/STAT3, and HIF‐1α levels. (F–H) Quantitative analysis of the bands reveals P‐JAK3/JAK3, P‐STAT3/STAT3, and HIF‐1α levels. (I) The protein levels of PTGS2, HMOX1, SERPINE1, and ICAM‐1. (J–M) Quantitative analysis of the bands reveals PTGS2, HMOX1, SERPINE1, and ICAM‐1 levels. # # #*p* < 0.001 as compared with Sham group; *p < 0.05, **p < 0.01, ***p < 0.001 as compared with MCAO group.

**FIGURE 8:**
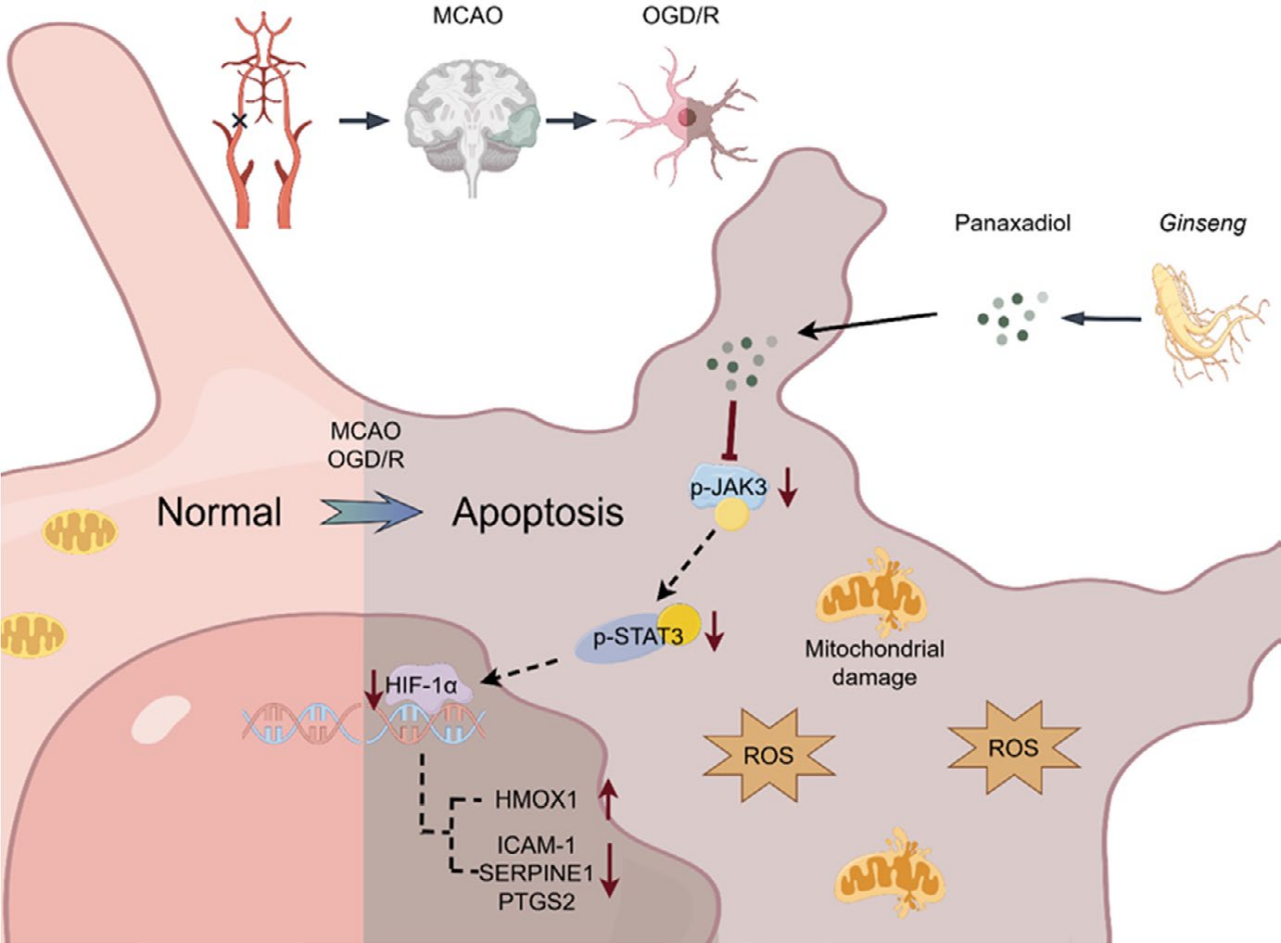
Graphical illustration of the drug action mechanism. (by Figdraw).

We examined the levels of pro‐inflammatory cytokines and antioxidant enzymes in the cerebral cortex of mice (Figure 6A–F). Compared to the sham groups, the levels of IL‐1β, IL‐6, TNF‐α, and MDA were significantly increased, and the levels of SOD and CAT were decreased in MCAO groups. PD also significantly inhibited the elevation of pro‐inflammatory cytokine and MDA levels and promoted the levels of SOD and CAT in mice. TUNEL staining was performed to detect neuronal apoptosis in the cerebral cortex of mice. Our study found that TUNEL fluorescence intensity was significantly higher in the MCAO group than in the sham group. Compared with the MCAO group, the fluorescence intensity in the PD groups was significantly reduced. This indicates that PD inhibited neuronal apoptosis in the cerebral cortex in a dose‐dependent manner (Figure 6G), and the expression of apoptosis‐associated proteins confirmed these results (Figure 7A–D). Additionally, the protein expression of PTGS2, SERPINE1, and ICAM‐1 was significantly elevated in the MCAO group compared to the sham group, whereas PD administration inhibited this elevation. The protein expression of HMOX1 was decreased in the MCAO groups, and PD significantly reversed this decrease (Figure 7I–M). Consistent with the in vitro experiments, the expressions of phosphorylated‐JAK3, phosphorylated‐STAT3, and HIF‐1α were significantly increased in MCAO groups (Figure 7E–H). Compared to the MCAO groups, the protein expressions of phosphorylated‐JAK3, phosphorylated‐STAT3, and HIF‐1α were mitigated by PD treatment in a dose‐dependent manner (Figures 7E–H and 8). These in vitro results further support the predicted hub targets from the network pharmacology analysis, confirming that PD reduced neuronal apoptosis in MCAO mice by inhibiting the JAK3/STAT3/HIF‐1α signaling pathway.

## 4 Discussion

CIS, the most common type of stroke, accounts for approximately 70% of all strokes [45]. With the growth in the elderly population, the incidence of ischemic stroke is gradually increasing [46, 47]. Alleviating CIRI is crucial to improve the success rate of CIS treatment. Traditional Chinese herbs and their active compounds offer promising treatment of CIS/CIRI, due to their effectiveness and minimal toxicity [48]. PD, an active component of ginseng, has good bioavailability and lipophilicity, allowing it to cross the blood–brain barrier and exert its pharmacological activities [49]. There have been a few reports showing the advantages of PD in the treatment of neurological diseases, such as Alzheimer's disease and Parkinson's disease. Studies have shown that a dose of 50 mg/Kg PD reduced neuronal synaptic damage in Alzheimer's disease by inhibiting the Fyn/GluN2B/CaMKIIα signaling pathway [43]. In addition, continuous administration of PD at concentrations of 25 and 50 mg/Kg for 2 weeks can improve PD symptoms by regulating intestinal flora and repairing damage to dopaminergic neurons [44]. Compound 14a, a PD derivative, exhibited significant neuroprotective properties, including apoptosis inhibition, prevention of tau hyperphosphorylation, modulation of beta‐amyloid, and reduction of ROS [25]. PD has also been identified as a potential neurogenic molecule capable of inducing neural stem cell differentiation into neurons [50]. Our results confirm that PD significantly improved cognitive functions in MCAO mice, reduced cerebral infarction area, and inhibited the loss of the neuronal marker NeuN. HE and Nissl staining also indicated significant neuroprotective effects of PD. These results suggest that PD is a promising neuroprotective candidate for CIS treatment, although its mechanism of action remains unclear. Consequently, we used network pharmacology analysis to further investigate the targets and mechanisms of PD in the treatment of CIS.

The JAK3/STAT3 signaling pathway is closely associated with CIRI [51, 52]. Following CIS, the phosphorylation levels of the JAK3/STAT3 proteins increase, promoting inflammatory responses and neuronal apoptosis. Studies have shown that curcumin mitigates brain damage caused by ischemia in rats. Its molecular mechanism is linked to the inhibition of the JAK2/STAT3 signaling pathway and the reduction of inflammatory responses [53]. Stachydrine can reduce neuronal apoptosis in brain tissue by decreasing the phosphorylation levels of STAT3 protein and, to some extent, delaying the pathological changes induced by CIRI in rats [54]. Our study also confirmed that, compared to the OGD/R or MCAO groups, the PD groups reduced the phosphorylation levels of JAK3/STAT3 proteins in HT22 cells and mouse brain tissue in a concentration‐dependent manner. This indicates that the inhibition of JAK3/STAT3 activation is a potential mechanism through which PD reduces inflammation and apoptosis. HIF‐1α is a transcription factor produced under hypoxic conditions, promoting an adaptive response to ischemia and hypoxia [55]. However, HIF‐1α expression has a dual role in protecting neurons and inducing apoptosis. Under hypoxic conditions, HIF‐1α promotes the transcription of downstream genes, regulating intracellular energy metabolism to enhance cellular adaptation to low oxygen levels [56]. However, under hypoxic conditions, HIF‐1α expression disrupts the balance between pro‐apoptotic and anti‐apoptotic factors, inducing the expression of pro‐apoptotic proteins and initiating apoptotic signaling pathways. Our results show that PD inhibits the expression of HIF‐1α, Bax, and caspase 3, suggesting that PD may suppress cell apoptosis and alleviate neuronal damage possibly by inhibiting the JAK3/STAT3/HIF‐1α pathway.

Our study further indicated that PD regulates the expression of hub genes and ameliorates CIRI. Prostaglandin‐endoperoxide synthase 2, (PTGS2) is a rate‐limiting enzyme in prostaglandin (PG) synthesis and is involved in the pathological and physiological processes of CIRI. Studies have shown that PTGS2 levels in the hippocampus of rats increase significantly after CIRI [57]. Inhibition of PTGS2 expression can significantly reduce brain infarct volume and alleviate motor coordination deficits caused by CIS. Our study found that PTGS2 expression was markedly elevated in the brain of mice in the MCAO group, whereas PD significantly suppressed its protein expression levels. HMOX1, an important antioxidant enzyme, reduces ROS levels and provides various endogenous protective functions, including antioxidant, anti‐inflammatory, and anti‐apoptotic effects [58]. Previous studies have demonstrated that many traditional Chinese medicines exert antioxidant effects through HMOX1, ameliorating oxidative stress in rat brain tissue [59, 60]. Therefore, HMOX1 is considered a protective marker for oxidative stress responses. Our results indicated that PD significantly upregulates HMOX1 protein expression in the MCAO group both in vitro and in vivo. SERPINE1, also known as plasminogen Activator Inhibitor‐1 (PAI‐1), is an important component of the plasma fibrinolytic system. An imbalance in this system plays a significant role in the pathogenesis of CIS [61]. This study found that PAI‐1 levels were significantly elevated in the MCAO model, possibly due to increased secretion from endothelial cell damage during cerebral ischemic infarction. PD significantly inhibited PAI‐1 protein levels in the brain tissues of MCAO mice. ICAM‐1, primarily expressed in vascular endothelial cells, increases during CIRI, further exacerbating brain tissue damage [62]. Research in MCAO rat models has demonstrated that ICAM‐1 expression mediates inflammatory responses, worsening brain tissue damage [63]. Our results indicate that PD inhibits ICAM‐1 expression, reducing CIRI, and improving neurological function in MCAO mice.

Although our experimental research has yielded promising results, large‐scale randomized controlled clinical trials are needed to validate the efficacy and safety of PD from basic research to clinical applications. Future research should focus on optimal clinical dosage, route of administration, and the potential integration of PD with conventional stroke treatments. Furthermore, exploring individualized treatment strategies will be an important direction in the future, especially through precision medicine. Tailored PD treatment plans for patients with different stroke types could maximize therapeutic benefits and enhance patient outcomes.

## 5 Conclusion

In summary, PD alleviates oxidative stress damage in mouse brain tissue caused by CIRI, by regulating the JAK3/STAT3/HIF‐1α signaling pathway (Figure 8). This mechanism involves enhancing antioxidant enzyme activity, reducing ROS production and inflammatory factor levels, and mitigating the impact of CIRI.

## Ethics Statement

The authors have nothing to report.

## Conflicts of Interest

The authors declare no conflicts of interest.

## Supporting information


Data S1.

## Data Availability

Data used to support the results of this study were included within the article and Supporting Information.
