# Supplementary figures and images for: Panaxadiol Attenuates Neuronal Oxidative Stress and Apoptosis in Cerebral Ischemia/Reperfusion Injury via Regulation of the JAK3/STAT3/HIF‐1α Signaling Pathway
Source: CNS Neurosci Ther. 2025 Feb 17;31(2):e70233. doi: 10.1111/cns.70233 (PMC11831195; doi:10.1111/cns.70233)

Figure 5E

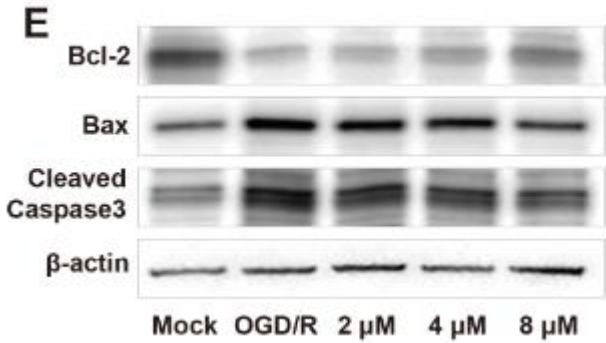

Marker (kda)

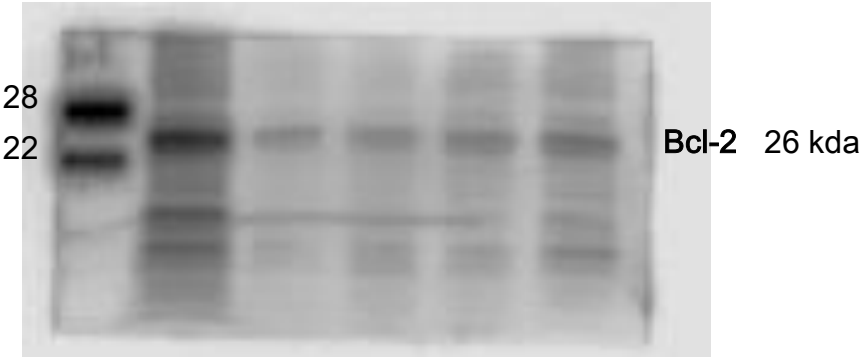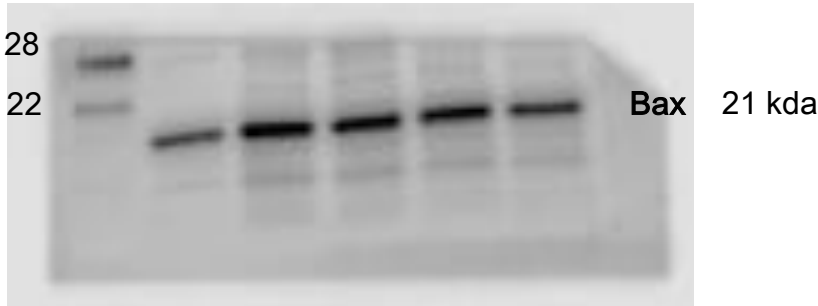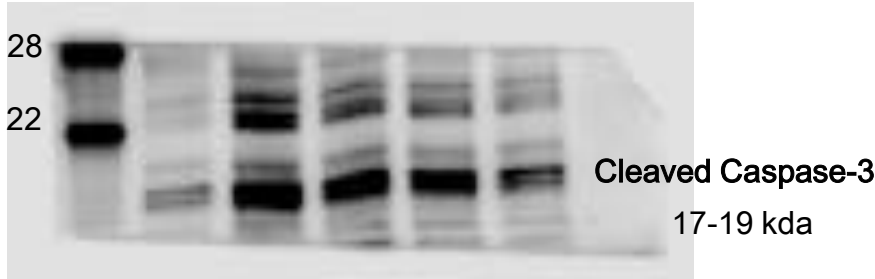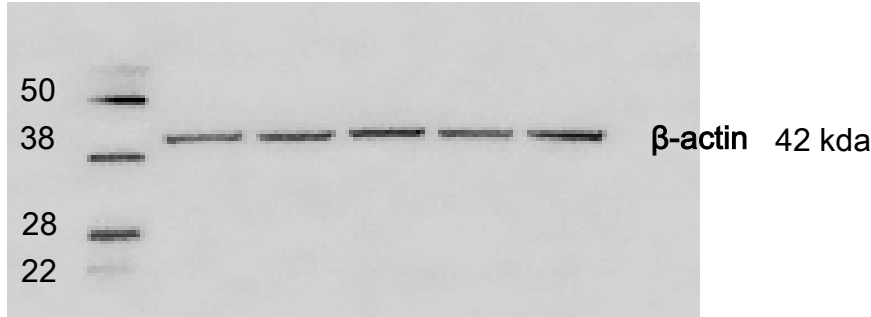

Figure 5I

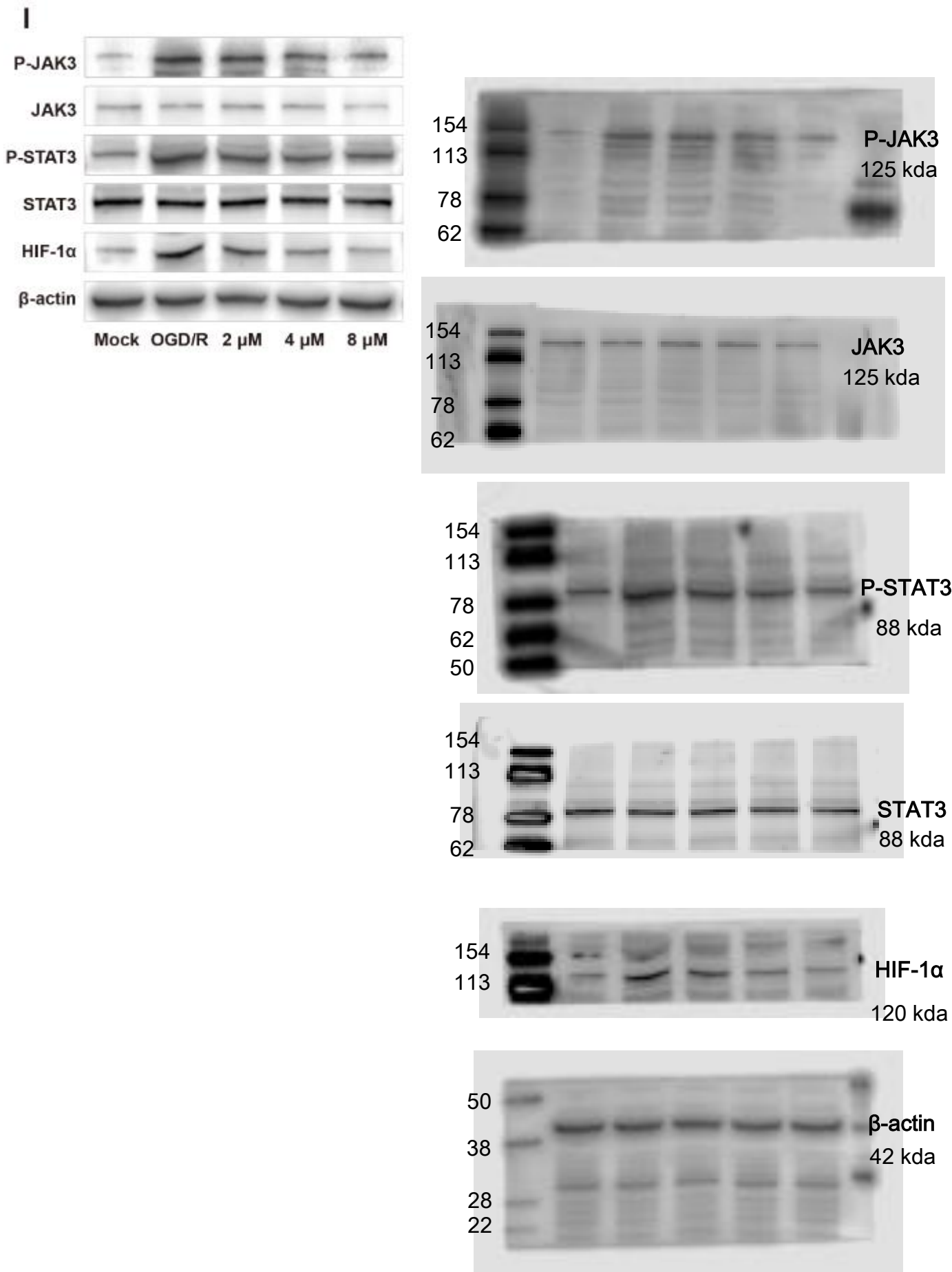

Figure 5M

M

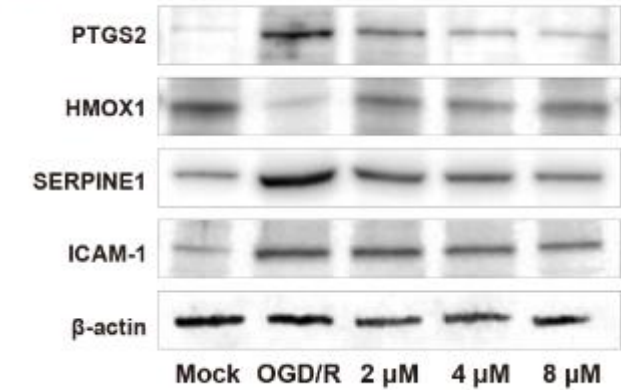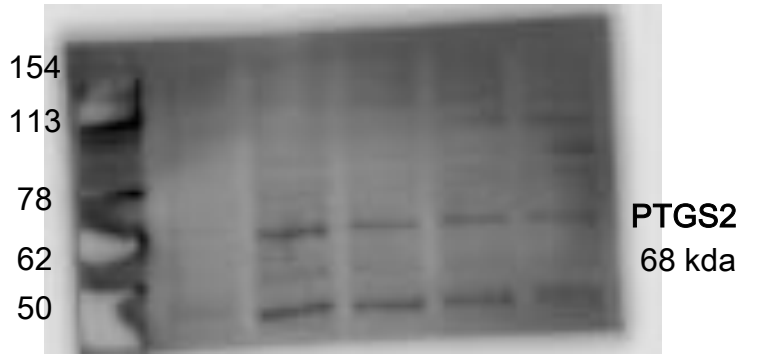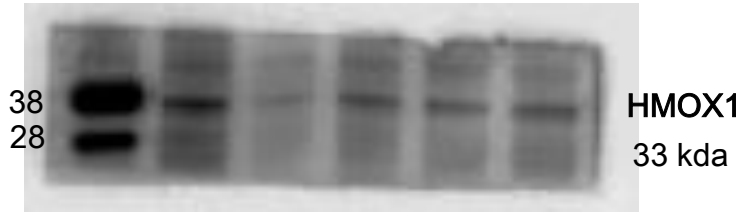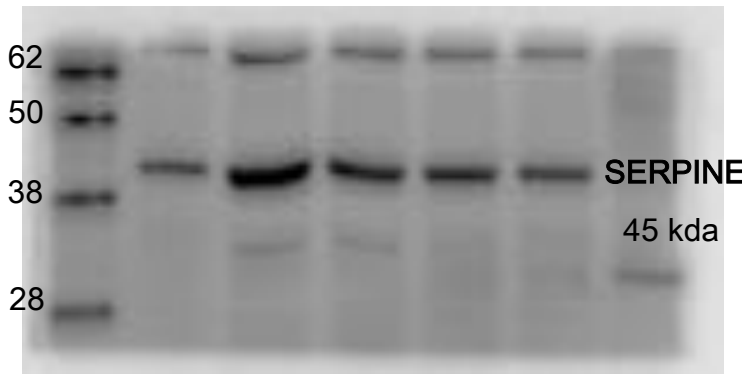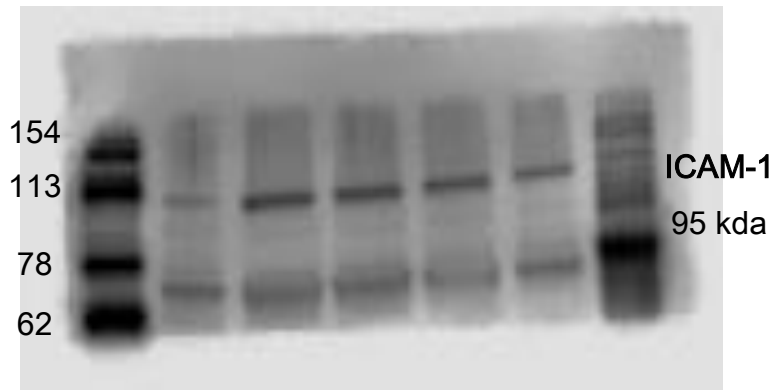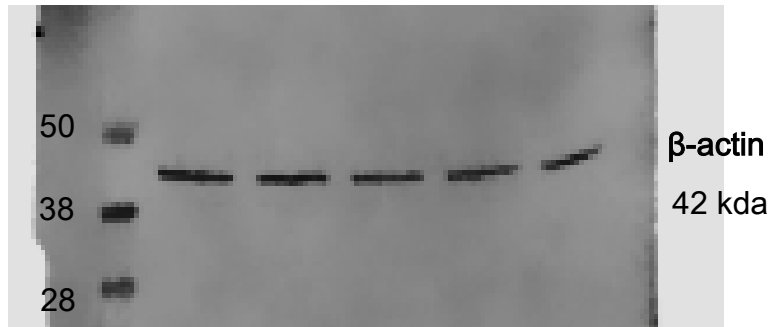

Figure 7A

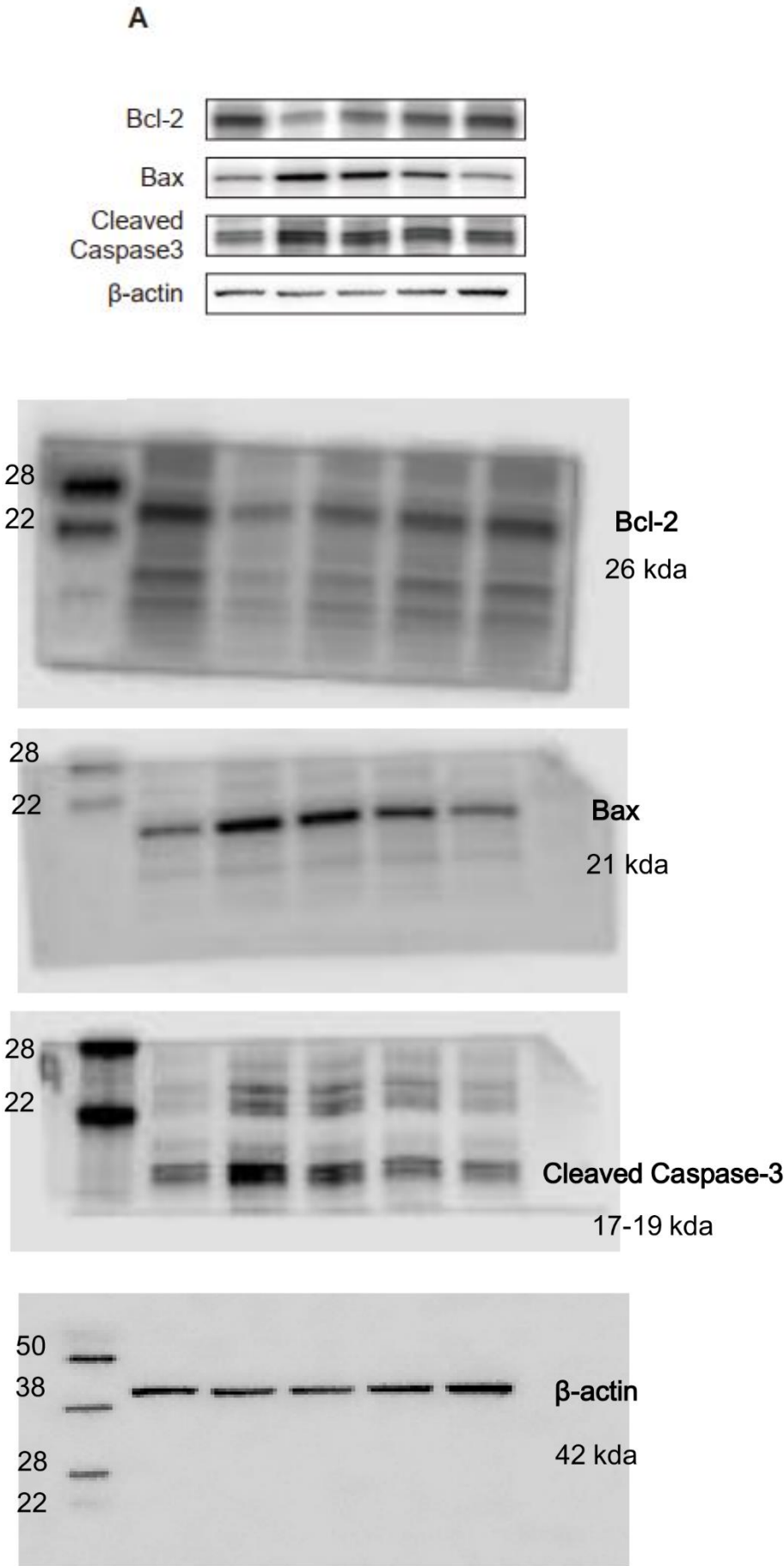

Figure 7E

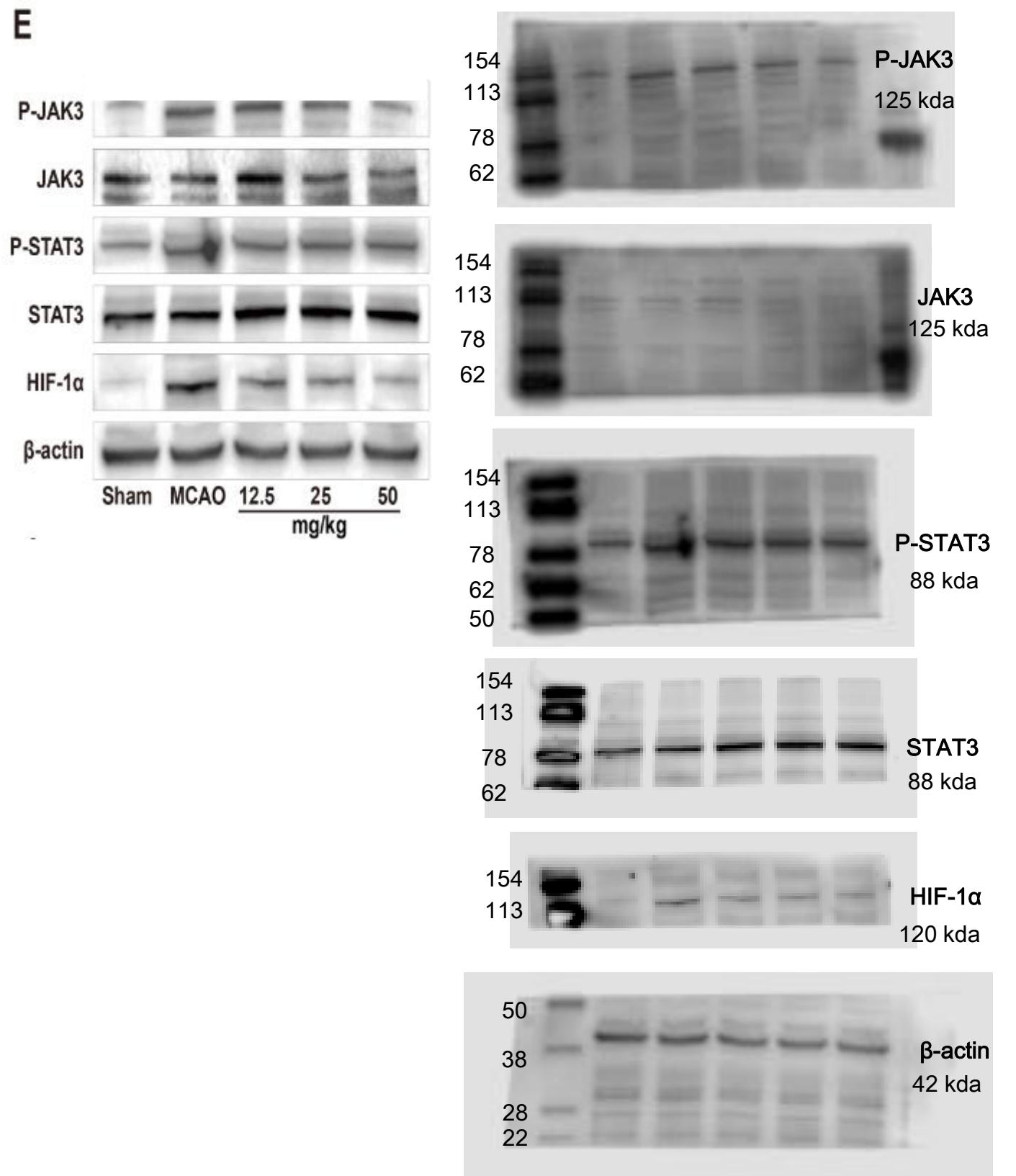

Figure 7l

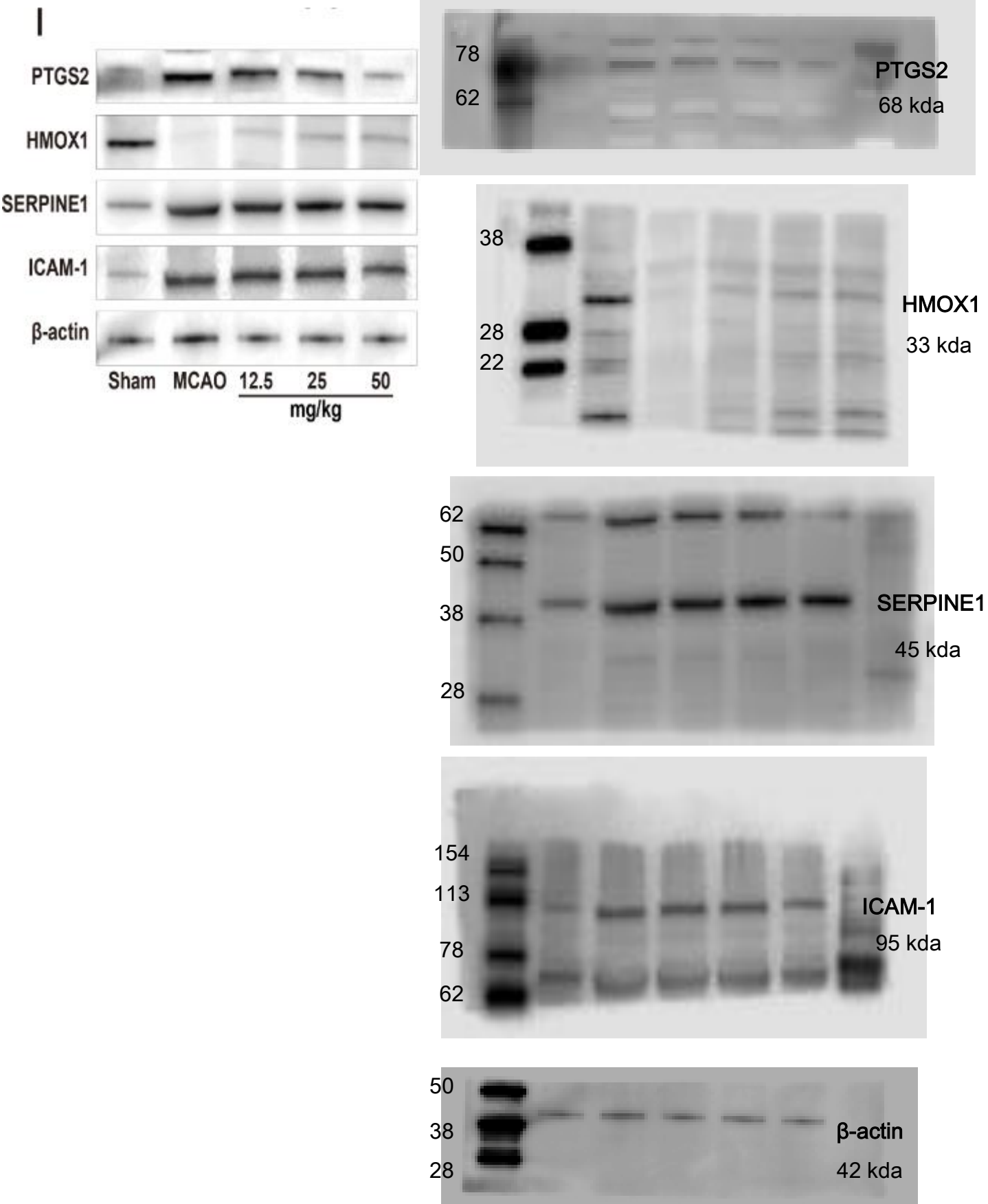

Supplement: Supplementary file 1 — Data S1. [file CNS-31-e70233-s001.pdf]
